# Entraining the Brain with Music and Synchronized Light to Improve Memory and Preserve Cognitive Function

**DOI:** 10.1093/geroni/igaf122.4286

**Published:** 2025-12-31

**Authors:** Edward Large, Ji Chul Kim, Psyche Loui

**Affiliations:** Oscillo Biosciences, Storrs, Connecticut, United States; Oscillo Biosciences, Willington, Connecticut, United States; Northeastern University, Boston, Massachusetts, United States

## Abstract

Worldwide at least 57 million individuals are currently living with Alzheimer’s disease (AD), and by 2050 nearly 13 million Americans are projected to have AD. Pharmacological interventions have not been successful in producing significant effects on memory, cognition and behavior, and all pharmaceuticals carry some risk of potentially dangerous side effects. Recent research suggests that rehabilitation of neural oscillations supporting memory and cognition may provide effective treatments for neurological disorders such as AD. However, there are significant challenges in developing non-invasive, feasible, effective and scalable methods for human intervention. We have developed an intervention to rehabilitate theta (4-8Hz) and gamma (30-50Hz) neural oscillations using self-selected music and synchronized light pulses. We are currently running a Phase I Clinical Trial (NCT05984524) in individuals with Mild Cognitive impairment (MCI) and early-stage AD. Our gamma-enhanced music-based intervention (gMBI) is administered in the patient’s home 30 minutes/day for 8 weeks; the control intervention includes listening to a health education podcast (the health-education intervention, HEI). Before and after the therapy patients undergo a battery of tests, including electroencephalography (EEG), structural magnetic resonance imaging (sMRI), diffusion tensor imaging (DTI), functional MRI (fMRI), blood draws, and tests of cognition and memory. Patient usage data in both conditions is monitored. We find that patients comply better with the treatment protocol than with the control protocol. After 8 weeks, neural oscillations are strengthened, hippocampal activation increases, memory improves, and cognitive function is preserved in the treatment group relative to controls. Patient recruitment is ongoing.

